# Supplementary material for: Involvement of Oxytocin and Progesterone Receptor Expression in the Etiology of Canine Uterine Inertia
Source: Int J Mol Sci. 2022 Nov 6;23(21):13601. doi: 10.3390/ijms232113601 (PMC9653816; doi:10.3390/ijms232113601)
Supplement: Supplementary file 1 [file ijms-23-13601-s001.zip › ijms-1994269-supplementary.pdf]

Supplementary Materials

Statistical analysis of OXTR and PGR staining intensity in IHC.

Table S1. Statistical analysis of OXTR-immunopositive staining. Comparison of layers: longitudinal versus circular myometrial layer differentiated in interplacental (IP) and uteroplacental (UP) tissue. Groups (primary uterine inertia, PUI; obstructive dystocia, OD) are presented individually and summarized.

	Group	*p*-Value (Wilcoxon Matched-Pairs Signed Rank Test)
IP	PUI	$p = 0.0156$
	OD	$p = 0.0156$
	PUI + OD	$p = 0.0001$
UP	PUI	$p = 0.5$
	OD	$p = 0.5$
	PUI + OD	$p = 0.0769$
IP + UP	PUI + OD	$p =< 0.0001$

Table S2. Statistical analysis of OXTR-immunopositive staining in interplacental (IP) tissue. Comparison of groups (primary uterine inertia, PUI, versus obstructive dystocia, OD) for each specific myometrial layer (longitudinal/circular) and the combined IP-Myoscore.

	Myometrial Layer	Groups for Comparison	*p*-Value (Mann–Whitney Test)
IP	Longitudinal	PUI vs. OD	$p = 0.2941$
		PUI-N vs. OD	$p = 0.1939$
IP	Circular	PUI vs. OD	$p = 0.4199$
		PUI-N vs. OD	$p = 0.8869$
IP	Myoscore	PUI vs. OD	$p = 0.5320$
		PUI-N vs. OD	$p = 0.2796$

Table S3. Effect of litter size in the primary uterine inertia (PUI) group on OXTR-immunopositive staining in IP tissue differentiating the myometrial layer (longitudinal/circular). PUI bitches with normal litter size (PUI-N, average litter size of the respective breed ±1 standard deviation) were compared with litters smaller (PUI-S, <–1 SD) or larger (PUI-L, >+1 SD) than the breed average.

	Myometrial Layer	Groups for Comparison	*p*-Value (Kruskal–Wallis Test)
IP	Longitudinal	PUI-S vs. PUI-N vs. PUI-L	$p = 0.2346$
IP	Circular	PUI-S vs. PUI-N vs. PUI-L	$p = 0.7273$
IP	Myoscore	PUI-S vs. PUI-N vs. PUI-L	$p = 0.655$

Statistical analysis of PGR staining intensity in IHC.

Table S4. Statistical analysis of PGR-immunopositive staining. Comparison of layers: longitudinal versus circular myometrial layer differentiating interplacental (IP) and uteroplacental (UP) tissue. Groups (primary uterine inertia, PUI; obstructive dystocia, OD) were presented individually and summarized.

	Group	*p*-Value (Wilcoxon Matched-Pairs Signed Rank Test)

IP	PUI	p = 0.625
	OD	p => 0.9999
	PUI + OD	p = 0.4531
UP	PUI	p = 0.5472
	OD	p => 0.9999
	PUI + OD	p = 0.6127
IP+ UP	PUI + OD	p = 0.8462

Table S5. Statistical analysis of PGR-immunopositive staining in interplacental (IP) samples. Comparison of groups (primary uterine inertia, PUI, versus obstructive dystocia, OD) depending on localization (interplacental, IP; uteroplacenta, UP) and specific myometrial layer (longitudinal/circular) or combined IP-Myoscore.

	**Myometrial Layer**	**Groups for Comparison**	***p*-Value (Mann–Whitney Test)**
IP	Longitudinal	PUI vs. OD	p = 0.1323
		PUI-N vs. OD	p = 0.097
IP	Circular	PUI vs. OD	p = 0.1161
		PUI-N vs. OD	p = 0.1455
IP	Myoscore	PUI vs. OD	p = 0.0716
		PUI-N vs. OD	p = 0.0626

Table S6. Effect of litter size on PGR-immunopositive staining in interplacental (IP) samples of the primary uterine inertia (PUI) group differentiating the myometrial layers (longitudinal/circular). PUI bitches with normal litter size (PUI-N, average litter size of the respective breed ±1 standard deviation) were compared with litters smaller (PUI-S, <−1 SD) or larger (PUI-L, >+1 SD) than the breed average.

	**Myometrial Layer**	**Groups for Comparison**	***p*-Value (Kruskal–Wallis Test Followed by Tukey's Multiple Comparisons)**
IP	Longitudinal	PUI-S vs. PUI-N vs. PUI-L	p = 0.0306
IP	Circular	PUI-S vs. PUI-N vs. PUI-L	p = 0.1984
IP	Myoscore	PUI-S vs. PUI-N vs. PUI-L	p = 0.0391
